# Patient acceptance of AI-assisted diabetic retinopathy screening in primary care: findings from a questionnaire-based feasibility study

**DOI:** 10.3389/fmed.2025.1610114

**Published:** 2025-09-10

**Authors:** Malene Krogh, Marie Germund Nielsen, Gabriela Byskov Petersen, Morten Sig Ager Jensen, Martin Bach Jensen, Henrik Vorum, Niels Henrik Bruun, Jette Kolding Kristensen

**Affiliations:** ^1^Center for General Practice at Aalborg University, Aalborg, Denmark; ^2^The Clinical Nursing Research Unit, Aalborg University Hospital, Aalborg, Denmark; ^3^Department of Health Science and Technology, Aalborg University, Aalborg, Denmark; ^4^Steno Diabetes Center Copenhagen, Copenhagen, Denmark; ^5^Department of Ophthalmology, Aalborg University Hospital, Aalborg, Denmark; ^6^Research Data and Biostatistics, Aalborg University Hospital, Aalborg, Denmark

**Keywords:** artificial intelligence, type 2 diabetes, diabetic retinopathy screening, primary care, patient acceptance, questionnaire development

## Abstract

**Aim:**

This feasibility study investigates patients' acceptance of AI-assisted diabetic retinopathy screening (DRS) in primary care.

**Method:**

Patients with type 2 diabetes from 12 primary care settings in Denmark underwent AI-assisted DRS as part of routine diabetes care and completed a questionnaire covering demographics, recent DRS, general health, mental well being, trust in physician, competence in diabetes self-care, distrust in AI, and acceptance of future DRS.

**Results:**

298 patients participated and completed the questionnaire. Acceptance of future AI-assisted DRS in primary care was higher than that of ophthalmologist-led screening, although patients still showed distrust toward AI. Findings indicated that greater competence in diabetes self-care was associated with higher acceptance of future AI-assisted DRS in primary care. Lower distrust in AI increased acceptance of future AI-assisted DRS in primary care, while higher distrust increased acceptance of ophthalmologist-led DRS.

**Conclusion:**

This study found that most patients accepted future AI-assisted DRS in primary care. Associations between acceptance and the factors examined are very small and may have limited or no clinical impact. Other factors, such as convenience of having DRS in primary care, may influence patient's acceptance.

## 1 Introduction

People with diabetes mellitus are at risk of developing various complications (1), including diabetic retinopathy (DR), which is a microvascular retinal complication that can lead to significant vision impairment and even blindness (2). A critical challenge associated with DR is its asymptomatic progression in the early stages, with most individuals experiencing no symptoms until the disease reaches its severe stages (3). Therefore, regular DR screening (DRS) is essential for detecting early retinal alterations and preventing vision loss (3, 4). Despite the proven effectiveness of systematic DRS in detecting sight-threatening alterations and reducing the risk of vision loss when combined with timely treatment (3, 4), suboptimal participation rates in DRS remains a challenge in most healthcare systems (5).

In Denmark, most patients with type 2 diabetes mellitus (T2D) are managed in general practice, while DRS is usually performed by private ophthalmologists (6). Screening intervals are individualized, ideally based on DR severity and diabetes regulation, and may range from 3 to 24 months (7). In 2023, 36% of patients with type 2 diabetes did not attend a screening within their recommended interval (6), and a Danish registry study showed that 26.6% had no registered DRS within a 6-year period (2013–2018) (8). Hence, there is a growing demand for new initiatives to improve participation in DRS (9).

Technological advancements in DRS, particularly the use of artificial intelligence (AI) for automated image grading, have enabled the outsourcing of screenings to primary care providers, improving accessibility and reducing workload for specialists. This AI-assisted approach has been tested internationally and has shown promising accuracy in prospective studies, achieving sensitivities of ≥87% and specificities of ≥84% (10–12). An important aspect of implementing new technologies in primary care is further to understand patients‘ perspectives. Patients can provide valuable insights based on their real-life experiences and their interacting with healthcare services offers important information about the impact of healthcare technologies (13–15), such as benefits or potential drawbacks. Patient' perspectives can uncover key factors that may not be fully captured in clinical or technical evaluations (13–15), but is crucial for patient's trust and acceptance of technologies. Incorporating patient perspectives may therefore offer a more holistic understanding of technology's effectiveness and its relevance in real-clinical contexts.

Research on patients' perspectives on AI-assisted DRS in primary care is increasing (16–20), and studies report similar results, with patients expressing positive views about AI-assisted DRS, with most indicating satisfaction and a willingness to use this method again in the future (16–18). However, some patients still express distrust regarding the prospect of AI replacing ophthalmologists (17, 20). A consistent finding in patient perspectives across various healthcare fields, including DRS, is the strong preference that AI should not operate independently, but only assist human judgment (20–25).

AI-assisted DRS is likely to form the foundation of most future DRS programs, as it has the potential to enhance efficiency, increase screening uptake, and reduce resource utilization (9). Given the differences in healthcare systems and patient populations, it is important to evaluate DRS in various settings. In Denmark, assessing the feasibility of this initiative and investigate patients' perspectives on DRS in primary care are crucial steps before broader implementation.

In this study, patients partook in AI-assisted DRS as part of routine diabetes care, and the aim of this feasibility study is to investigate their acceptance of AI-assisted DRS in primary care and identify potential factors influencing their acceptance through a developed questionnaire.

## 2 Method

### 2.1 Setting and participants

Participants in this cross-sectional feasibility study were recruited from 12 general practice clinics in Denmark between March 1, 2022, and December 1, 2023. Eligible patients were identified by clinic staff based on the following inclusion criteria: a diagnosis of T2D, age between 18 and 70 years, proficiency in Danish, no blindness in either eye, and the ability to attend a follow-up ophthalmologic DRS. In cases where the fundus examination could not be completed (e.g., due to small pupils or camera malfunction), the patient was excluded from the study and did not complete the questionnaire. In line with the feasibility nature of the study, no pre-study power calculation was conducted. Instead, each clinic was instructed to recruit as many eligible patients as possible within a predefined 4-week intervention period. Based on the expected number of T2D patients per clinic, estimated from national data, it was assumed that each clinic could recruit ~30 patients during the intervention period. This number was considered sufficient, to ensure adequate response data and to gain preliminary insight into patients' perspectives on the screening domains. However, in some clinics, recruitment proved challenging, primarily due to long travel distances to the affiliated ophthalmology clinic. To facilitate participation, the DRS follow-up criterion was therefore removed for patients recruited from the final two clinics. Apart from the defined inclusion and exclusion criteria, no further measures were implemented to control for potential selection bias.

### 2.2 AI-assisted DRS

AI-assisted DRS was performed using a non-mydriatic fundus camera (FundusScope, Rodenstock, Germany) with a 45° field of view. The camera is equipped with a 12-megapixel sensor and captures high-resolution retinal images (4,096 × 3,072 pixels) using an integrated LED flash. As patients did not receive dilating eye drops, a 20-s pause was implemented between image captures to reduce the impact of pupillary constriction caused by the flash.

The AI software (RetinaLyze System; RetinaLyze A/S, Hørsholm, Denmark) (26) was used to analyze the retinal images. Utilizing a Support Vector Machine learning algorithm, the software detects and classifies visible retinal changes by identifying red lesions in the image. The number of red lesions detected determines the result, and results are presented using a color-coded system: green indicates no alterations, yellow few alterations, red implies several alterations (>3 lesions), and gray indicates insufficient image quality. The AI analysis is completed in ~15 s. RetinaLyze has been available on the market since 2013 and was provided free of charge for use during the study period.

The AI output was not used as a replacement for clinical grading but served as an initial screening tool. In 10 of the 12 clinics, all patients were referred for a follow-up ophthalmologic examination with a collaborating ophthalmologist, regardless of AI result. In the last two clinics, only patients with a yellow or red AI result were advised to consult their own ophthalmologist. The AI results were therefore not used to make formal clinical decisions but may have influenced referral recommendations in these two clinics.

### 2.3 Procedure

Recruited patients attended their diabetes consultation in primary care and took part in a DRS performed by their usual diabetes care provider. For most patients, the DRS was conducted by their diabetes nurse, but for a few patients, the DRS was performed by their general practitioner or a biomedical laboratory scientist. All patients were informed that the analysis of their fundus images in primary care would be performed solely by the AI system, and that their diabetes care provider would not assess the images. The DRS was completed within a few minutes, after which patients were verbally informed of their results. Some patients were already aware of their DR status from previous screenings, while for others, this was their first DRS. Finally, all patients were asked to complete a purpose-developed questionnaire on a tablet (Huawei MediaPad T3 10). If needed, patients could request technical assistance from their diabetes care provider.

All patients, except those from the last two recreated clinics, were later to attent the follow-up screening at a participating ophthmologist clinic.

### 2.4 Questionnaire development

**Figure 1:**
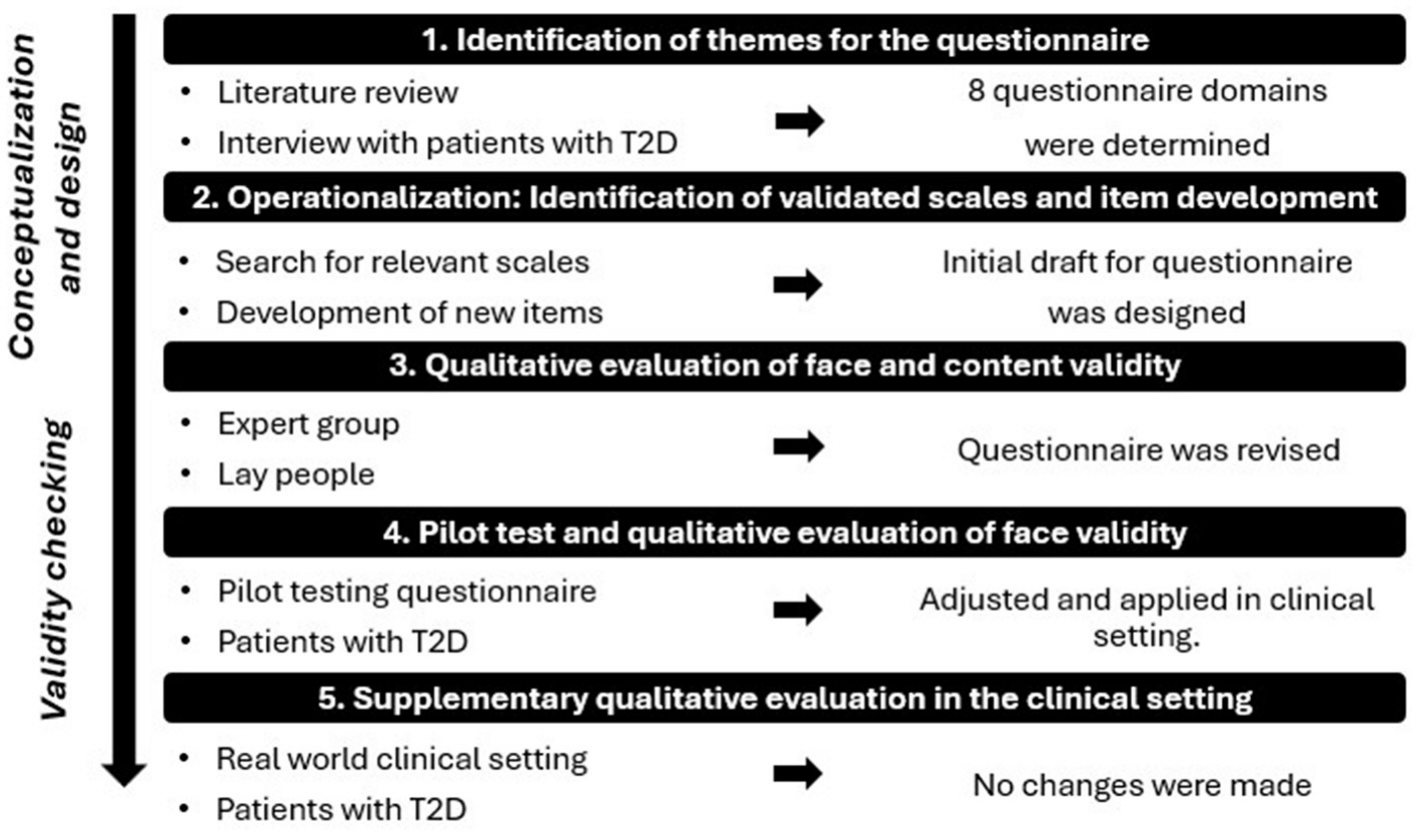
Illustration of the questionnaire development phases, simplifying a complex and iterative process. T2D: Type 2 Diabetes.

As no relevant questionnaire existed at the initiation of this study to assess factors influencing patients' acceptance of AI-assisted DRS in primary care, a new questionnaire was developed specifically for this purpose. The development process involved identification of key themes through literature review and qualitative interviews, followed by multiple rounds of cognitive testing, field evaluations, and iterative refinement (see Figure 1).

#### 2.4.1 Step 1: identification of themes for the questionnaire

To identify themes for the questionnaire, a literature review was conducted (27), focusing on patient attitudes and perspectives toward AI in diabetes care, later broadened to perspectives on AI in general healthcare due to lack of available studies specially on diabetes care. No formal method was used to extract themes; rather, recurring topics identified across the reviewed literature were used to guide the selection of questionnaire domains. Additionally, data from 20 semi-structured interviews with individuals diagnosed with T2D, aged 35–78, further informed the identification of themes. The interviews were conducted as part of a related Ph.D. study (9).

This led to the inclusion of eight key domains in the questionnaire: Demography, Latest DRS, General Health, Mental Well Being, Competence in Diabetes Self-Care, Trust in Physician, Distrust in AI in DRS and Acceptance of Future DRS.

#### 2.4.2 Step 2: operationalization: identification of validated scales and scale development

Relevant scales were identified through a search of patient-reported literature specific to T2D, as well as through questionnaire databases (28, 29). Validated scales were incorporated into the questionnaire in original or shortened forms for the domains: Demography, General Health, Mental Well Being, and Competence in Diabetes Self-Care. Items were developed for the domains: Latest DRS, Trust in Physician, Distrust in AI in DRS, and Acceptance of Future DRS. The development of new scales and items was based on similar questionnaires from various healthcare fields, with items being tailored to fit the DRS context (27). The relevance of items was carefully assessed, including phrasing and selection of suitable response options (27, 30). For an explanation and insight into all incorporated scales, please see Supplementary material 1.

The questionnaire combines scales with varying response categories: 5-, 6-, and 7-point Likert scales for most domains, while Demography, General Health and Latest DRS use alternative response formats (See questionnaire overview in Supplementary material 1).

The questionnaire was implemented into the software: Research Electronic Data Capture (31), where it was designed in a single-page layout.

#### 2.4.3 Step 3: qualitative evaluation of content and face validity

##### 2.4.3.1 Expert group

Face and content validity were established by an expert group (27), consisting of seven experts, including researchers, clinical professors, general practitioners, and an ophthalmologist all possessing extensive knowledge in the fields of diabetes, primary care, DRS, and questionnaire development.

##### 2.4.3.2 Lay people

Face validity was assessed through a qualitative evaluation with six laypeople (October 2021). This group consisted of four men and two women, aged 54.5 ± 13.9, with diverse educational backgrounds and varying levels of familiarity with AI—ranging from unfamiliar to well-aware with general AI use, including some knowledge of its application in image analysis. The aim was to evaluate how lay people evaluated questionnaire understanding, scale appropriateness, and tablet functionality, while also measuring completion time. After participants completed the questionnaire, MK conducted a “Double Interview” (27), where participants discussed selected items from the questionnaire, explaining their answers and their understanding of the questions, providing insights into how they interpreted the items.

#### 2.4.4 Step 4: pilot test and qualitative evaluation of face validity

To assess face validity, scale appropriateness, and acceptability of the questionnaire, a pilot test was conducted with two patients with type 2 diabetes in a primary care setting (December 2021). The participants were a 74-year-old man (2 years with T2D, limited knowledge of AI) and a 72-year-old woman (7 years with T2D, unfamiliar with AI). Both first underwent a simulated DRS performed by MK, followed by completion of the questionnaire on a tablet. MK then conducted cognitive interviews using the “Double Interview” technique (27).

Adjustments were made based on the pilot test. Following this, the questionnaire was applied in the feasibility study. As data collection progressed, a supplementary qualitative evaluation was conducted at one clinic to evaluate patients' understanding of the questionnaire in a real-world clinical setting.

#### 2.4.5 Supplementary qualitative evaluation in the clinical setting

During the clinical setup in one selected practice, a supplementary qualitative evaluation was conducted to explore how patients understood and interpreted the questionnaire in the context of the actual screening setup. After completing the questionnaire immediately following their AI-assisted DRS, five women and five men with type 2 diabetes (mean age 60.6 ± 7.3 years, with limited knowledge or unfamiliar with AI) participated in cognitive interviews using the “Double Interview” technique (27). Based on this supplementary evaluation, no modifications were deemed necessary. The questionnaire was therefore used in its current form across all 12 participating clinics, and data collected with the current version was included in the final analysis of the feasibility study.

### 2.5 Data analysis

Baseline characteristics, including age, sex, marital status, previous DRS, latest DRS, and general health, were presented as means and standard deviations (SD) for continuous data, and as frequencies and percentages for categorical data. Each Likert-scale item is described by the frequency and percentage of each response category, along with the item's mean and standard deviation. Composite scores are presented as averages of their items. The reliability is assessed using Cronbach's alpha. Differences in average composite scores are estimated using OLS regression with robust variance estimation and are reported with 95% confidence intervals and *p*-values. Responses with missing data on individual items were excluded from the corresponding analyses. Stata 18 was used for data management and statistical analysis.

## 3 Results

### 3.1 Patient characteristics

A total of 298 patients completed the questionnaire after undergoing AI-assisted DRS in primary care. Table 1 summaries patient characteristics.

**Table 1:** Patient characteristics.

**Variables**	**Summary**	**Missing/N (Pct)**
*n* (%)	298 (100.0)	0/298 (0.0)
**Sex**, ***n*** **(%)**		
Female	128 (43.2)	
Male	168 (56.8)	2/298 (0.7)
Age, mean (sd)	61.9 (8.4)	2/298 (0.7)
**Marital status**, ***n*** **(%)**		
In a relationship^a^	229 (76.8)	
Single	59 (19.8)	
Other^b^	8 (2.7)	2/298 (0.7)
**Previous DRS**, ***n*** **(%)**		
Yes	236 (79.5)	
No	59 (19.9)	
Don't know	2 (0.7)	1/298 (0.3)
**Latest DRS**, ***n*** **(%)**		
Within the last year	188 (79.7)	
Within the last 2 years	31 (13.1)	
More than 2 years ago	10 (4.2)	
Don't know	5 (2.1)	
Not relevant to me	2 (0.8)	62/298 (20.8)
**General health**, ***n*** **(%)**		
Poor	4 (1.4)	
Fair	62 (21.0)	
Good	146 (49.5)	
Very good	70 (23.7)	
Excellent	13 (4.4)	3/298 (1.0)

^a^Covers both “relationship” and “married”.

^b^Covers both “other” and “prefer not to disclose”. Missing/N (Pct) refers to the number and percentage of participants with missing responses on each variable.

### 3.2 Reliability of questionnaire

All scales demonstrated high reliability, with Cronbach's alpha values above 0.8. The scores were as follows: Mental well being = 0.888, Trust in Physician = 0.873, Competence in Diabetes Self-care = 0.887, and Distrust in AI in DRS = 0.867. For further details see Supplementary material 2.

### 3.3 Distrust in AI in DRS

Table 2 presents the mean scores and distribution of patient responses regarding distrust in AI in DRS. The table includes the mean score and SD for each item across the entire sample, as well as the total counts and percentages for each response category. Higher mean scores reflect higher distrust in AI in DRS, except for two positively formulated items on AI (“*AI will replace ophthalmologists in the future*” and “*AI can prevent errors*”) where higher scores indicate low distrust in AI. Overall, the mean scores show that patients hold some distrust toward AI, particularly regarding its ability to replace ophthalmologists (mean = 3.29, SD = 0.99) and concerns about AI independently analyzing images without human oversight (mean = 2.91, SD = 1.01). The item “*Ophthalmologist detects more*” received the highest distrust (mean = 3.48, SD = 0.89), while “*Concerned AI ignores feelings” has the lowest distrust (2.50, SD* = *1.04)*.

**Table 2:** Distrust in AI in DRS: mean scores and response distribution.

**Abbreviated items**	**Mean^a^**	**sd**	**Don't know**	**Strongly disagree**	**Disagree**	**Neither agree nor disagree**	**Agree**	**Strongly agree**
AI can never replace ophthalmologist's experience	3.29	0.99	33 (11.2)	11 (3.7)	41 (13.9)	100 (33.9)	82 (27.8)	28 (9.5)
Ophthalmologist detects more through experience	3.48	0.89	30 (10.2)	3 (1.0)	30 (10.2)	102 (34.6)	98 (33.2)	32 (10.8)
Worried about AI analysis alone	2.91	1.01	22 (7.5)	21 (7.1)	74 (25.2)	98 (33.3)	66 (22.4)	13 (4.4)
AI not ready for image analysis	2.62	0.92	33 (11.2)	29 (9.8)	87 (29.5)	107 (36.3)	33 (11.2)	6 (2.0)
AI will replace ophthalmologists in the future^b^	2.79	1.07	26 (8.8)	36 (12.2)	70 (23.7)	87 (29.5)	67 (22.7)	9 (3.1)
Won't blindly trust AI	3.23	0.97	20 (6.8)	13 (4.4)	47 (16.0)	94 (32.1)	102 (34.8)	17 (5.8)
AI should only double-check	3.20	1.01	20 (6.8)	14 (4.7)	56 (19.0)	87 (29.5)	97 (32.9)	21 (7.1)
Concerned AI ignores feelings	2.50	1.04	28 (9.5)	48 (16.3)	90 (30.6)	82 (27.9)	38 (12.9)	8 (2.7)
Unsure how AI analyses images	2.87	0.96	37 (12.5)	24 (8.1)	59 (20.0)	108 (36.6)	60 (20.3)	7 (2.4)
Prefer ophthalmologist even if AI is better	3.01	1.05	19 (6.5)	21 (7.1)	70 (23.8)	87 (29.6)	79 (26.9)	18 (6.1)
AI use risks personal data	2.56	1.09	34 (11.5)	44 (14.9)	89 (30.2)	81 (27.5)	31 (10.5)	16 (5.4)
AI can prevent errors^b^	3.38	0.84	42 (14.4)	7 (2.4)	16 (5.5)	123 (42.3)	82 (28.2)	21 (7.2)

^a^min: 0 – max 5. Percentages of respondents are shown in parentheses. Overall, higher scores indicate higher distrust toward AI in DRS. However, items marked with ^b^ are positively formulated on AI, and higher mean score indicates lower distrust toward AI in DRS.

The two positively formulated items on AI demonstrated low distrust toward AI, where the statements “*AI will replace ophthalmologists in the future*” showed a mean score of 2.79, SD = 1.07. Similar, the item “*AI can prevent errors*” received a relatively high agreement (mean = 3.38, SD = 0.84), being the item with the second highest mean score in the scale, indicating lower distrust in AI. The table reveals that a substantial proportion of patients selected “Neither agree nor disagree,” with percentages ranging from 27.5 to 42.3%.

### 3.4 Acceptance of future DRS

Table 3 presents the mean scores and distribution of patient responses regarding acceptance of future DRS. The table includes mean score and SD for each item across the entire sample, and the total counts and percentages for each response category. The results indicate a higher acceptance for future DRS in primary care compared to acceptance of future ophthalmologist DRS (mean = 3.6 & 2.78, respectively). The response categories for both items were distributed across the scale, with most responses falling under “Agree” for acceptance of future DRS in primary care (47.8%) and “Neither agree nor disagree” for acceptance of future ophthalmologist DRS (38%).

**Table 3:** Acceptance of future DRS: mean scores and response distribution.

**Abbreviated items**	**Mean^a^**	**sd**	**Don't know**	**Strongly disagree**	**Disagree**	**Neither agree nor disagree**	**Agree**	**Strongly agree**
Accept PC DRS	3.60	0.88	17 (5.8)	8 (2.7)	19 (6.4)	79 (26.8)	141 (47.8)	31 (10.5)
Accept ophthalmologist DRS	2.78	0.98	18 (6.1)	25 (8.5)	82 (27.8)	112 (38.0)	45 (15.3)	13 (4.4)

^a^min: 0 – max 5. Percentages of respondents are shown in parentheses. Higher scores indicate greater acceptance.

PC, primary care; DRS, diabetic retinopathy screening.

### 3.5 Association between domains

Table 4 presents mean scores, effects, 95% CI and *p* values based on the regression analysis. The analysis identified a significant association between patients' acceptance of future DRS in primary care and their competence in diabetes self-care (*p* = 0.04). Specifically, higher levels of confidence in diabetes self-care were associated with increased acceptance of future DRS in primary care (Effect size = 0.13, Lower and upper 95% CI = 0.01–0.25). A significant finding (*p* = 0.00) also shows that decrease in distrust in AI in DRS increase acceptance of future DRS in primary care (Effect size = −0.03, Lower and upper 95% CI = −0.04 to −0.02). Furthermore, there was a significant association between distrust in AI in DRS and their acceptance of future ophthalmologist DRS (*p* = 0.00), demonstrating that higher distrust in AI in DRS increases patients' acceptance of future ophthalmologist DRS (Effect size = 0.04, Lower and upper 95% CI = 0.02–0.05).

**Table 4:** Association between domains.

**Depended variable**	**Independent variable**	**Mean^a^**	**Effect**	**Lower 95% CI**	**Upper 95% CI**	***p* Value**
Accept PC DRS	Previous DRS (No)^b^	3.67				
	Previous DRS (Yes)	3.59	−0.08	−0.32	0.15	0.47
	General health (Poor)^b^	3.50				
	General health (Fair)	3.63	0.13	−1.00	1.27	0.82
	General health (Good)	3.53	0.03	−1.09	1.15	0.96
	General health (Very good)	3.75	0.25	−0.88	1.38	0.66
	General health (Excellent)	3.58	0.08	−1.22	1.39	0.90
	Well being total (0–100)	3.60	0.00	−0.00	0.01	0.16
	Trust in physician total (0–100)	3.60	0.01	−0.00	0.02	0.06
	Self-confidence in care (0–7)	3.60	0.13	0.01	0.25	0.04
	Distrust AI total (0–100)	3.60	−0.03	−0.04	−0.02	0.00
Accept ophthalmologist DRS	Distrust AI total (0–100)	2.78	0.04	0.02	0.05	0.00

^a^Mean of dependent variable. ^b^Effect, 95% CI and *p* Values are not applicable for the reference and therefore omitted.

PC, primary care; DRS, diabetic retinopathy screening.

For a detailed insight into the results obtained on the scale for Mental Well Being, Competence in Diabetes Self-Care, and Trust in Physician, refer to Supplementary material 2.

## 4 Discussion

### 4.1 Statement of principal findings

This study provides new insights into patients' acceptance of AI-assisted DRS in primary care. A key finding in this comprehensive study is that most patients accept future AI-assisted screenings in primary care, while the majority remained neutral about future traditional ophthalmologist DRS. Despite acceptance, patients still express distrust toward AI in DRS, and have greater trust in ophthalmologists. Most patients preferred that AI be used solely to double-check the ophthalmologist's assessment, while some believed AI could never match ophthalmologists‘ experience. However, patients acknowledged that AI could potentially reduce diagnostic errors and even replace ophthalmologists in the future.

The results are interesting but should be interpreted with caution. The findings suggest that competence in diabetes self-care and distrust in AI may influence acceptance of future DRS. However, the effect sizes for all statistically significant associations were very small, and this feasibility study was not based on a formal power calculation. Accordingly, it remains uncertain whether, or to what extent, these results are clinically relevant.

### 4.2 Principle findings in relation to other studies

Patients have previously expressed satisfaction with and a willingness to use DRS in primary care settings (16–18), however, some patients still hesitate toward AI replacing ophthalmologists (16–18, 32). These findings align with the present study, where patients expressed greater trust in ophthalmologists and preferred AI to serve only as a second opinion. Despite distrust in AI, the acceptance of future screenings remained highest in primary care. This could potentially suggest that the advantages of screening in primary care may outweigh the distrust toward AI. Although we cannot definitively explain the acceptance, primary care may offer greater convenience for patients. Factors such as distance, long waiting list for appointments, and the need for eye drops at ophthalmologist practices serve as barriers to current DRS attendance (32, 33), and T2D patients have previously endorsed the idea of outsourcing DRS to primary care, emphasizing the potential for increased convenience in diabetes care (32). Further, satisfaction with diabetes care in primary care is influences by patients' trust in their physician (34, 35), and in this study, patients reported high trust in their diabetes care provider. While physician trust did not directly affect patients' acceptance of future DRS, high physician trust may enhance their satisfaction with DRS in primary care. Patients with T2D and healthcare professionals have suggested that DRS in primary care could improve attendance (18, 32, 36, 37). There may therefore be benefits to implementing DRS in primary care, such as increased convenience and satisfaction with diabetes care and potentially higher DRS attendance.

Interest in trust in AI systems has grown in recent years (38–40), as trust is widely recognized as essential for acceptance (40). We measured distrust, but acknowledge that trust and distrust are conceptually complex—sometimes treated as distinct constructs, while other times as opposite ends of the same dimension (38, 39, 41, 42). The study that inspired our scale (24) did not define distrust explicitly, but we interpreted it as the inverse of trust. This should be considered when interpreting the findings of the article.

In the context of AI, trust depends not only on the system's functionality, but also on its transparency and social factors (39, 40). While users of traditional technologies often have more control over the technology's functions, some AI systems are more unpredictable due to their “black-box” nature, where decision-making processes may not be transparent and easily understood (39, 40, 43).

### 4.3 Implications for policymakers

The implementation of AI-assisted tools is on the political agenda (44), which will require a significant shift in existing healthcare practices. However, adoption of new health technologies can evoke fear for patients (45), and in the case of AI-assisted DRS, a lack of understanding of AI and concerns about its future reliability remain key barriers to patient acceptance (19). At the moment, AI is still unfamiliar to many, and its clinical applications are not yet well understood by patients (19, 20, 23). Patients have expressed that reassurance from healthcare professionals and clear step-by-step explanations of AI functionality in DRS can help build confidence and trust (19). Therefore, if AI-assisted DRS is to be implemented in primary care, it is crucial to address patients‘ concerns and support them during this transition, ensuring that distrust and lack of knowledge do not become barriers to participation. If not, these issues may hinder the successful adoption of AI-assisted DRS, potentially reducing patient engagement and compromising the effectiveness of the screening program. Policymakers should ensure that patients' needs are acknowledged and consider how healthcare professionals can play an active role in educating patients about the role of AI in healthcare.

### 4.4 Strengths and weaknesses of the study

A strength of this study is that all patients completed the questionnaire after having participated in an AI-assisted DRS in primary care. This means they had firsthand experience with DRS and specific aspects addressed in the questionnaire.

However, the developed questionnaire introduces several limitations. First, at the time of development, only one questionnaire on AI in healthcare was found to be relevant (24), and it ultimately served as inspiration for the scale Distrust in AI in DRS. This scale is predominantly composed of negatively worded statements about AI, with only two items phrased in a positive manner. This asymmetry in tone may have influenced response patterns, as all items were likely to elicit agreement. As a result, the overall score for distrust might not fully reflect participants' actual attitudes. Future refinements of the scale should consider formulating items in a more neutral tone. Furthermore, an examination of participants' responses reveals that a substantial proportion selected 'neither agree nor disagree.' This may indicate ambiguity in item interpretation, uncertainty regarding the topic, or a general lack of strong opinion. Such response patterns could suggest a need for further refinement of scale items to enhance clarity and ensure that participants can meaningfully differentiate between response options.

Second, the Trust in Physician scale includes both positive and negative formulated items. This required patients to alternate between agreeing and disagreeing to respond accurately, which may have introduced response bias among inattentive respondents.

Third, the initial pilot testing included only two patients with T2D and six laypeople without T2D. This small sample may not have captured all potential issues with item clarity or relevance. Given the small number of participants in the initial validation, we considered it necessary to conduct a supplementary evaluation to investigate the questionnaire's clarity. This evaluation was not conducted in the first practice recruited, which would have been preferable. Since no changes were made, questionnaire data was used across all clinics. The evaluation was conducted with 10 patients, all from the same practice. This may limit the generalizability of the findings due to potential contextual bias—particularly in a study involving multiple clinics with diverse patient populations.

Finally, this study was limited to Danish general practice settings, included participants aged below 70 years, and utilized a single AI-assisted DRS system. These factors may limit generalizability to other healthcare systems, regions, or older populations. Also, adjustments made to the inclusion criteria in the final two clinics due to recruitment difficulty can also have introduced selection bias and affected the results.

### 4.5 Future research

Future studies with sufficient statistical power are needed to validate these preliminary findings and determine whether the identified factors meaningfully influence patient acceptance of AI-assisted DRS. Research should also examine other possible determinants, such as socioeconomic status or convenience of having DRS in primary care settings, to better understand variation in acceptance. To improve generalizability, future studies should include multi-center and cross-regional designs and test the applicability of findings across different healthcare systems and patient populations. In addition, further refinement and validation of the questionnaire in broader and more diverse samples is needed to ensure its validity and relevance across settings.

Although this study did not focus on ethical implications, concerns such as responsibility for potential AI errors, patient safety, and data privacy are important aspects to consider when implementing AI-assisted DRS. These issues warrant further investigation and should be addressed in future research.

Lastly, further research is needed to gain a deeper understanding of how patients can be supported during the transition to AI-assisted healthcare. Qualitative studies involving both patients and key stakeholders could help identify effective strategies for facilitating this shift. Such insights may be relevant not only to DRS but also to other healthcare domains where AI is expected to play an increasingly significant role.

## 5 Conclusion

This study found patient acceptance for future AI-assisted DRS in primary care, but distrust toward AI remained. Caution is needed when drawing conclusions about the associations between acceptance of DRS and the tested factors, as the effects of these factors are small and may have no or limited clinical relevance. The acceptance of AI-assisted DRS is complex and may be influenced by additional factors such as the convenience of receiving screening in primary care.

## Data Availability

The datasets presented in this article are not readily available because “The dataset contains personal identification numbers (CPR numbers), phone numbers, and email addresses, which can identify participants. Part of the data can be made available upon request, but identifiable information will be removed beforehand.” Requests to access the datasets should be directed to Malenekrogh@dcm.aau.dk.
