# Supplementary material for: Patient acceptance of AI-assisted diabetic retinopathy screening in primary care: findings from a questionnaire-based feasibility study
Source: Front Med (Lausanne). 2025 Sep 10;12:1610114. doi: 10.3389/fmed.2025.1610114 (PMC12457666; doi:10.3389/fmed.2025.1610114)
Supplement: Supplementary file 1 [file Table_1.DOCX]

**Supplementary Material 1 – Questionnaire Overview and Argument for Questionnaire Domains**

This document outlines the rationale behind the selected domains included in the questionnaire. While many of the scales are previously validated, some domains were specifically developed to align with the study's objectives. The process of creating new domains is also detailed in this document.

**Tabel 2A: Questionnaire Overview**

| NO | Domain | Number of items | Response categories | Origins | Developed |
| --- | --- | --- | --- | --- | --- |
| 1 | Demographic | 2 | 9 and 5 | Danish PRO |  |
| 2 | Previous DRS | 1 + 3 | 3 + 3, 5 and 6 |  | Yes |
| 3 | General health | 1 | 5 | SF-12 |  |
| 4 | Mental Well-being | 5 | 6-point likert scale | WHO-5 |  |
| 5 | Trust in Physician | 10 | 6-point likert scale |  | Yes |
| 6 | Competence in diabetes self-care | 3 | 8-point likert scale | Perceived Competence for Diabetes Scale |  |
| 7 | Distrust in AI in DRS | 12 | 6-point likert scale |  | Yes |
| 8 | Future DRS | 3 | 6-point likert scale |  | Yes |

DRS: Diabetic Retinopathy Screening

**Project ID** (1 item)

The patient was required to fill out their unique project ID, so questionnaire data could be linked with other data.

­­­­­­­­­­­­­­­­­­­­­­­­­­­­­­­­­­­­­­­­­­­­­­­­­­­­­­­­­­­­­­­­­­**Demographic information** (2 items)

To gather demographic information, two items were included to assess patients' education level and marital status.

**Previous visits to eye doctor** (1 item (plus 3 items))

Interviews and existing literature revealed that transportation to the ophthalmology practice can be a barrier for some individuals in participating in DRS, due to factors such as distance, limited transportation options, or financial constraints (1). To gather information on patients' previous visits to the ophthalmologist, one item was included to ask whether they had previously seen an ophthalmologist as part of their diabetes care. If the patient selected "yes" three additional items were presented to further investigate the details of their previous visit. These items included questions about the interval since latest visit, whether eye drops were given during that visit, and how patients travelled to and from the ophthalmologist's practice.

Item development

The visit item regarding last visit to an ophthalmologist practice was developed with inspiration from an item used in the Danish PRO "Livet med diabetes 2019" (2). The original item was modified during development to clarify whether "having your eyes checked" referred to an eye doctor or an optician, as it caused confusion. The last three items were developed by the expert group using clear and understandable language.

**General health** (1 item)

We wanted information about patients’ general health to get a better understanding of the individual, and how general health potentially influence acceptance of future DRS.

Item in questionnaire

With the number of items in mind, only a single question about self-rated general health was implemented in the questionnaire. This item appears in other questionnaires and measure general health, such as SF-12(3) and PROMIS Global-10 Health(4).

**Mental Well-being** (5 items)

The prevalence of depression among adults with type 2 diabetes is higher than that in the general population (5,6). Depression has been shown to be associated with poor adherence to diabetes self-care, including DRS (7). To gain a more comprehensive understanding, we sought to assess their mental well-being in relation to patients’ acceptance of future DRS in primary care.

Scale in questionnaire

The scale chosen for the questionnaire was the WHO-5, which is a brief, generic scale designed to measure mental well-being, comprising five simple questions (8,9). This scale evaluates mental well-being over the past two weeks. The scale is available Danish, and the WHO-5 has been used extensively within diabetes research where a low WHO-5 score may have a substantial negative impact upon diabetic control (10). We chose the WHO-5 to measure mental well-being due to its concise format, straightforward language, and ease of understanding across different age groups (10,11)

**Trust in physician** (10 items)

Trust is an important component of any relationship, particularly the one between patients and physicians. Trust in one’s physician is known to influence several health-related factors including the quality of the patient-provider interaction and patient satisfaction with care (12,13). Previous studies conducted among adults with diabetes showed that increased trust in one’s physician has been associated with better self-care (14,15). Furthermore, a review showed that trust in physician has been associated with retinopati attendance (16). We therefore wanted to investigate if trust in physician would affect patients’ acceptance of future AI-assisted DRS in primary care. However, to our knowledge no Danish validated scale matched our purpose.

Development of the scale in questionnaire

We developed a new scale to measure patients trust towards their diabetes health care professional in primary care with inspiration from the “Trust in Physician Scale” (Anderson & Dedrick, 1990). The original scale is composed of 11 items, scored on a 5-point Likert scale ranging from strongly disagree to strongly agree.

When developing new items, we tried to make the Danish wording close to the original scale while ensuring an easy understandable language. The original Trust in Physician Scale was transferred to a Word document, serving as a foundation for creating a new Danish version with a slightly different aim. As the new scale was only inspired by the original, the wording was adjusted to suit the context of this study. For example, when the original scale used the term ‘doctor’, we changed it to ‘diabetes healthcare provider’. Also, when the original scale referred to ‘medical care’, we used “diabetes care”.

The first version of the new scale was made by MK, and soon after the first draft, several meetings was held with the expert group to discuss the developed scale and adjust. It was decided to reduce the scale to 10 items and therefore exclude item 8 from the original scale. During the qualitative pilot testing, this item was perceived as challenging to understand by several participants. Following a thorough evaluation by the expert group, it was determined that the item should be removed from the questionnaire.

**Competence in diabetes self-care** (3 items)

Patients with chronic medical conditions, such as type 2 diabetes, must engage in self-management to achieve optimal health outcomes. Research has shown that patients' perceived competence in managing their condition is closely linked to self-care behaviours (17). In particular, greater self-efficacy beliefs have been associated with improved health outcomes across various conditions (18).

Findings from the conducted interviews revealed that patients' ability to manage self-care in daily life influenced their likelihood of scheduling an appointment for DRS. Several patients reported that they "did not have the time" or "had not prioritized scheduling an appointment." Notably, patients with lower self-care abilities expressed a positive attitude toward integrating DRS into routine diabetes consultations in primary care. This observation led us to explore patients’ competence in self-care to better understand how it may affect their acceptance of future AI-assisted DRS conducted within primary care settings.

Scale in questionnaire

To measure the patients’ competence in their diabetes self-care, we used the Danish version of the Perceived Competence for Diabetes Scale(PCDS) (19). The scale assesses the degree to which people with diabetes feel they can manage the aspects of diabetes care (19,20). A low score may indicate that the patient is struggling with diabetes self-care and may require additional patient education, self-care, and support.

**Distrust towards AI in DRS** (12 items)

At the time of the development of this questionnaire, no well-documented literature on the topic of patients’ trust or distrust towards AI in DRS existed. However, distrust in AI had been a central topic in various healthcare fields. The literature indicates that patients generally hold positive attitudes toward the use of AI for diagnostic and treatment planning purposes. However, studies also suggest that people have a distrust towards AI, believing AI should not be relied upon independently, but rather serve as a support tool to enhance human judgment (21–23). Privacy security has also been a concern for many regarding the implementation of AI in health care (21,24). We therefore found it important to investigate patients distrust towards AI in DRS.

Development of the scale in questionnaire

We developed a new scale to measure patients distrust towards AI in DRS with inspiration from a validated scale investigating patients’ acceptance of AI in radiology (24). The scale was inspired by the domain regarding distrust and accountability which originally consists of 15 items. When developing new items, we tried to make the Danish wording close to the original scale while ensuring an easy understandable language. The original scale was transferred to a Word document, serving as a foundation for creating a new Danish version with a slightly different aim. As the new scale was only inspired by the original, the wording was adjusted to suit the context of this study. For example, when the original scale used the term ‘doctor’ or ‘radiologist’, we changed the wording to ‘eye-doctor’. Also, when the original scale referred to ‘evaluating scans’, the formulation was always changes to “analysing photos”. The original scale alternates between the terms "AI" and "computer" when referring to artificial intelligence. A key concern during the development of the new scale was determining the most appropriate terminology for AI. As AI remains a relatively new concept, some respondents may be unfamiliar with the term. We were concerned that certain respondents might not fully understand what "AI" entails. However, we also recognized that using alternative terms such as "computer," "machine," or "automated analysis" could fail to accurately capture the concept of AI as intended.

This issue was thoroughly discussed with the expert group and discussed with eight individuals with varying levels of familiarity with AI. Following extensive deliberations, it was decided to retain the term "artificial intelligence" in the new scale. To ensure clarity for respondents, a brief description of AI and its application in DRS was included in the introductory text of the survey. Additionally, an asterisk was placed next to the term "artificial intelligence" each time it appeared in items, referring respondents to a concise explanation positioned above the scale. During the actual DRS process, healthcare professionals also provided participants with a verbal explanation of AI and how it is applied in the DRS context.

The first version of the new scale was made by MK, and soon after the first draft, several meetings was held with the expert group to discuss the developed scale and adjust. The final version of the scale consisted of 12 items, based on relevance of items and with the number on items in mind.

**Acceptance of Future DRS** (3 items)

We wanted to explore patients' acceptance of future AI-assisted DRS in primary care, as well as their acceptance of future traditional ophthalmologist-led DRS.

Scale in questionnaire

No validated questionnaire was available and new items was developed. The three items were inspired by a questionnaire investigating patients’ acceptance towards telemedicine (25), especially the item “I wouldn’t want the telemedicine to alter my traditional way of using health care services”. The development process for the three items followed the same procedure as that used for the previous scale, however the three items was not as close to the inspirational items. For example, all new items had a positive tone, and for every item it was specified how and where future eye screenings could be performed.

**Additional items**

At the end of the questionnaire, patients were required to provide their name, CPR number, phone number, and email address. This information was required to ensure that patients could be contacted for follow-up DRS appointments with an ophthalmologist. CPR number was also used to determine gender and age of the patients.

**References**

1. Lawrenson JG, Graham-Rowe E, Lorencatto F, Rice S, Bunce C, Francis JJ, et al. What works to increase attendance for diabetic retinopathy screening? An evidence synthesis and economic analysis. NIHR Journals Library; 2018. https://doi.org/10.3310/hta22290

2. Diabetesforeningen. Livet med diabetes 2019. Diabetesforeningens Barometerundersøgelse 2019, https://diabetes.dk/media/jbtpmofg/rapport_livet-med-diabetes_2019.pdf [Accessed 6 March 2025)

3. Jenkinson C, Layte R, Jenkinson D, Lawrence K, Petersen S, Paice C, et al. A shorter form health survey: can the SF-12 replicate results from the SF-36 in longitudinal studies? J Public Health Med. 1997;19(2):179–86. https://doi.org/10.1093/oxfordjournals.pubmed.a024606

4. Hays RD, Bjorner JB, Revicki DA, Spritzer KL, Cella D. Development of physical and mental health summary scores from the patient-reported outcomes measurement information system (PROMIS) global items. Qual Life Res. 2009;18(7):873–80. https://doi.org/10.1007/s11136-009-9496-9

5. Wang F, Wang S, Zong QQ, Zhang Q, Ng CH, Ungvari GS, Xiang YT. Prevalence of comorbid major depressive disorder in Type 2 diabetes: a meta-analysis of comparative and epidemiological studies. Diabet Med. 2019;36(8):961-969. https://doi.org/10.1111/dme.14042.

6. Roy T, Lloyd CE. Epidemiology of depression and diabetes: a systematic review. J Affect Disord. 2012;142 Suppl:S8-21. https://doi.org/10.1016/S0165-0327(12)70004-6

7. Sumlin LL, Garcia TJ, Brown SA, Winter MA, García AA, Brown A, et al. Depression and adherence to lifestyle changes in type 2 diabetes: a systematic review. Diabetes Educ. 2014;40(6):731–44. https://doi.org/10.1177/0145721714538925

8. Hall T, Krahn GL, Horner-Johnson W, Lamb G, Rehabilitation Research and Training Center Expert Panel on Health Measurement. Examining functional content in widely used Health-Related Quality of Life scales. Rehabil Psychol. 2011;56(2):94–9. https://doi.org/10.1037/a0023054

9. Hajos TRS, Pouwer F, Skovlund SE, Den Oudsten BL, Geelhoed-Duijvestijn PHLM, Tack CJ, et al. Psychometric and screening properties of the WHO-5 well-being index in adult outpatients with Type 1 or Type 2 diabetes mellitus. Diabet Med J Br Diabet Assoc. 2013;30(2):e63-69. https://doi.org/10.1111/dme.12040

10. Topp CW, Østergaard SD, Søndergaard S, Bech P. The WHO-5 Well-Being Index: a systematic review of the literature. Psychother Psychosom. 2015;84(3):167–76. https://doi.org/ 10.1159/000376585

11. Lucas-Carrasco R, Allerup P, Bech P. The Validity of the WHO-5 as an Early Screening for Apathy in an Elderly Population. Curr Gerontol Geriatr Res. 2012;2012:171857. https://doi.org/10.1155/2012/171857

12. Benkert R, Peters RM, Clark R, Keves-Foster K. Effects of perceived racism, cultural mistrust and trust in providers on satisfaction with care. J Natl Med Assoc. 2006;98(9):1532–40. PMCID: PMC2569718

13. Sayed Ahmed HA, Abdelsalam NE, Joudeh AI, Abdelrahman AG, Eldahshan NA. Association of treatment satisfaction and physician trust with glycemic control among primary care patients with type 2 diabetes in Egypt. Diabetol Int. 2023;15(1):67–75. https://doi.org/10.1007/s13340-023-00653-x

14. White RO, Osborn CY, Gebretsadik T, Kripalani S, Rothman RL. Health Literacy, Physician Trust, and Diabetes-related Self-care Activities in Hispanics with Limited Resources. J Health Care Poor Underserved. 2013;24(4):1756–68. https://doi.org/10.1353/hpu.2013.0177

15. Ahmed Balla S. Satisfaction with Diabetes Services at Primary Care Level, Khartoum State, Sudan. Am J Health Res. 2016;4(5):127. https://doi.org/ 10.11648/j.ajhr.20160405.13

16. Lawrenson JG, Graham-Rowe E, Lorencatto F, Burr J, Bunce C, Francis JJ, et al. Interventions to increase attendance for diabetic retinopathy screening. Cochrane Database Syst Rev. 2018;1(1):CD012054. https://doi.org/10.1002/14651858.CD012054.pub2

17. Wallston KA, Rothman RL, Cherrington A. Psychometric Properties of the Perceived Diabetes Self-Management Scale (PDSMS). J Behav Med. 2007;30(5):395–401. https://doi.org/10.1007/s10865-007-9110-y

18. Bandura A. Self-efficacy: The exercise of control. New York, NY, US: W H Freeman/Times Books/ Henry Holt & Co; 1997. ix, 604 p.

19. Zoffmann V, Lauritzen T. Guided self-determination improves life skills with type 1 diabetes and A1C in randomized controlled trial. Patient Educ Couns. 2006;64(1–3):78–86. https://doi.org/ 10.1016/j.pec.2005.11.017

20. Williams GC, Freedman ZR, Deci EL. Supporting autonomy to motivate patients with diabetes for glucose control. Diabetes Care. 1998;21(10):1644–51. https://doi.org/10.2337/diacare.21.10.1644

21. Lennox-Chhugani N, Chen Y, Pearson V, Trzcinski B, James J. Women’s attitudes to the use of AI image readers: a case study from a national breast screening programme. BMJ Health Care Inform. 2021;28(1):e100293. https://doi.org/10.1136/bmjhci-2020-100293

22. Lennartz S, Dratsch T, Zopfs D, Persigehl T, Maintz D, Große Hokamp N, et al. Use and Control of Artificial Intelligence in Patients Across the Medical Workflow: Single-Center Questionnaire Study of Patient Perspectives. J Med Internet Res. 2021 Feb 17;23(2):e24221. https://doi.org/

23. Nelson CA, Pérez-Chada LM, Creadore A, Li SJ, Lo K, Manjaly P, et al. Patient Perspectives on the Use of Artificial Intelligence for Skin Cancer Screening: A Qualitative Study. JAMA Dermatol. 2020;156(5):501. https://doi.org/10.1001/jamadermatol.2019.5014

24. Ongena YP, Haan M, Yakar D, Kwee TC. Patients’ views on the implementation of artificial intelligence in radiology: development and validation of a standardized questionnaire. Eur Radiol. 2020;30(2):1033–40. https://doi.org/10.1007/s00330-019-06486-0

25. Kamal SA, Shafiq M, Kakria P. Investigating acceptance of telemedicine services through an extended technology acceptance model (TAM). Technol Soc. 2020;60:101212. https://doi.org/10.1016/j.techsoc.2019.101212.
