# Supplementary material for: Patient acceptance of AI-assisted diabetic retinopathy screening in primary care: findings from a questionnaire-based feasibility study
Source: Front Med (Lausanne). 2025 Sep 10;12:1610114. doi: 10.3389/fmed.2025.1610114 (PMC12457666; doi:10.3389/fmed.2025.1610114)
Supplement: Supplementary file 2 [file Table_2.DOCX]

**Supplementary Material 3 – Additional Results**

**Well-being**

**Table 3A: Well-being: Reliability (Cronbach’s alpha)**

| Abbreviated items | Item-rest | Mean interim | alpha |
| --- | --- | --- | --- |
| Feeling happy and upbeat | 0.763 | 0.597 | 0.856 |
| I have felt calm and relaxed | 0.737 | 0.609 | 0.862 |
| I have felt active and energetic | 0.741 | 0.607 | 0.861 |
| Feeling refreshed this morning | 0.661 | 0.645 | 0.879 |
| Enjoying my days fully | 0.739 | 0.608 | 0.861 |
| Test scale |  | 0.613 | 0.888 |

**Tabel 3B: Well-being: Mean Scores and Response Distribution**

| Abbreviated items | mean | sd | None | Some | Less than half | More than half | Most | All |
| --- | --- | --- | --- | --- | --- | --- | --- | --- |
| Feeling happy and upbeat | 4.68 | 0.96 | 1 (0.3) | 13 (4.4) | 23 (7.8) | 40 (13.5) | 184 (62.2) | 35 (11.8) |
| I have felt calm and relaxed | 4.47 | 1.09 | 3 (1.0) | 19 (6.4) | 30 (10.1) | 59 (19.9) | 153 (51.7) | 32 (10.8) |
| I have felt active and energetic | 3.89 | 1.29 | 15 (5.1) | 35 (11.8) | 50 (16.9) | 82 (27.7) | 95 (32.1) | 19 (6.4) |
| Feeling refreshed this morning | 4.00 | 1.32 | 15 (5.1) | 34 (11.5) | 40 (13.5) | 80 (27.0) | 101 (34.1) | 26 (8.8) |
| Enjoying my days fully | 4.45 | 1.12 | 4 (1.4) | 20 (6.8) | 25 (8.4) | 75 (25.3) | 133 (44.9) | 39 (13.2) |

Trust in Physician

**Tabel 4A: Trust in Physician: Reliability (Cronbach’s alpha)**

| Abbreviated items | Item-rest | Mean interim | alpha |
| --- | --- | --- | --- |
| I doubt my physician care about me (r) | 0.317 | 0.453 | 0.882 |
| My physician puts my needs first | 0.530 | 0.418 | 0.866 |
| I follow my physicians advise | 0.635 | 0.401 | 0.858 |
| My physician speaks the truth | 0.660 | 0.397 | 0.856 |
| I seek a second opinion beyond my physician (r) | 0.563 | 0.412 | 0.863 |
| I trust my physician’s judgement | 0.683 | 0.394 | 0.854 |
| My physician isn’t doing enough (r) | 0.572 | 0.411 | 0.863 |
| My physician is well-qualified | 0.676 | 0.395 | 0.855 |
| My physician alerts me to treatment errors | 0.754 | 0.383 | 0.848 |
| My physician do not keep confidentiality in my treatment (r) | 0.552 | 0.414 | 0.864 |
| Test scale |  | 0.408 | 0.873 |

(r): items are negatively formulated towards trust in physician

**Tabel 4B: Trust in Physician: Mean Scores and Response Distribution**

| Abbreviated items | mean | sd | Don’t know | Strongly disagree | Disagree | Neither agree nor disagree | Agree | Strongly agree |
| --- | --- | --- | --- | --- | --- | --- | --- | --- |
| I doubt my physician care about me (r) | 2.02 | 1.31 | 11 (3.7) | 145 (49.3) | 60 (20.4) | 29 (9.9) | 26 (8.8) | 23 (7.8) |
| My physician puts my needs first | 4.17 | 0.81 | 5 (1.7) | 6 (2.0) | 2 (0.7) | 31 (10.5) | 148 (50.2) | 103 (34.9) |
| I follow my physicians advise | 4.32 | 0.73 | 2 (0.7) | 4 (1.4) | 3 (1.0) | 13 (4.4) | 147 (49.8) | 126 (42.7) |
| My physician speaks the truth | 4.22 | 0.68 | 5 (1.7) | 1 (0.3) | 4 (1.4) | 24 (8.1) | 161 (54.6) | 100 (33.9) |
| I seek second opinion beyond my physician(r) | 1.83 | 1.00 | 9 (3.1) | 132 (45.1) | 98 (33.4) | 31 (10.6) | 16 (5.5) | 7 (2.4) |
| I trust my physician’s judgement | 4.29 | 0.75 | 3 (1.0) | 4 (1.4) | 3 (1.0) | 19 (6.4) | 145 (49.2) | 121 (41.0) |
| My physician isn’t doing enough (r) | 1.75 | 1.01 | 9 (3.1) | 149 (50.7) | 91 (31.0) | 19 (6.5) | 19 (6.5) | 7 (2.4) |
| My physician is well-qualified | 4.34 | 0.73 | 7 (2.4) | 3 (1.0) | 3 (1.0) | 17 (5.8) | 135 (45.9) | 129 (43.9) |
| My physician alerts me to treatment errors | 4.35 | 0.65 | 5 (1.7) | 1 (0.3) | 3 (1.0) | 13 (4.4) | 149 (50.5) | 124 (42.0) |
| My physician don’t keep confidentiality in my treatment (r) | 1.40 | 0.69 | 14 (4.8) | 192 (65.3) | 72 (24.5) | 9 (3.1) | 6 (2.0) | 1 (0.3) |

(r): items are negatively formulated towards trust in physician. Higher mean scores indicate greater trust in physician.

**Competence in Diabetes Self-care**

**Tabel 5A: Reliability (Cronbach’s alpha) of scale: Competence in Diabetes Self-Care**

| Abbreviated items | Item-rest | Mean interim | alpha |
| --- | --- | --- | --- |
| Confident in ability | 0.750 | 0.762 | 0.865 |
| Handles routine care efficiently | 0.767 | 0.740 | 0.850 |
| Manages diabetes challenges well | 0.822 | 0.669 | 0.802 |
| Test scale |  | 0.724 | 0.887 |

**Tabel 5B: Competence in Diabetes Self-Care: Mean Scores and Response Distribution**

| Abbreviated items | mean | sd | Don’t know | Disagree | Somewhat disagree | Neither agree nor disagree | Somewhat agree | Agree | Strongly agree |
| --- | --- | --- | --- | --- | --- | --- | --- | --- | --- |
| Confident in ability | 5.91 | 0.94 | 3 (1.0) | 5 (1.7) | 2 (0.7) | 12 (4.1) | 46 (15.6) | 157 (53.2) | 70 (23.7) |
| Handles routine care efficiently | 6.08 | 0.89 | 1 (0.3) | 3 (1.0) | 6 (2.0) | 6 (2.0) | 22 (7.5) | 170 (57.6) | 87 (29.5) |
| Manages diabetes challenges well | 5.95 | 0.96 | 2 (0.7) | 5 (1.7) | 6 (2.0) | 7 (2.4) | 36 (12.2) | 167 (56.6) | 72 (24.4) |

**Distrust in AI**

**Table 6A: Distrust in AI in DRS: Reliability (Cronbach’s alpha)**

| Abbreviated items | Item-rest | Mean interim | alpha |
| --- | --- | --- | --- |
| AI can never replace ophthalmologist’s experience | 0.518 | 0.356 | 0.859 |
| Ophthalmologist detects more through experience | 0.559 | 0.351 | 0.856 |
| Worried about AI analysis alone | 0.650 | 0.341 | 0.850 |
| AI not ready for image analysis | 0.662 | 0.339 | 0.850 |
| AI will replace ophthalmologist in the future (r) | 0.399 | 0.371 | 0.866 |
| Won’t blindly trust AI | 0.611 | 0.345 | 0.853 |
| AI should only double-check | 0.531 | 0.355 | 0.858 |
| Concerned AI ignores feelings | 0.481 | 0.361 | 0.861 |
| Unsure how AI analyses images | 0.521 | 0.356 | 0.859 |
| Prefer ophthalmologist even if AI is better | 0.601 | 0.346 | 0.854 |
| AI use risks personal data | 0.604 | 0.346 | 0.853 |
| AI can prevent errors (r) | 0.466 | 0.363 | 0.862 |
| Test scale |  | 0.352 | 0.867 |

(r): items are positively formulated towards trusting AI in DRS. AI: Artificial intelligence
